# A Decade of Oncolytic Virotherapy in Pediatric Cancers: A Systematic Review of Safety, Immune Awakening, and Emerging Efficacy

**DOI:** 10.7759/cureus.101744

**Published:** 2026-01-17

**Authors:** Amani S BinSharhan, AlJouhrah M AlAbdullah, Shouq F Alabdullatif, Yara Y Aboushark, Noura M Alateeq, Wala A Alhajjaj, Hajer O Alrabea, Fajr Saeedi

**Affiliations:** 1 College of Medicine, Al-Maarefa University, Riyadh, SAU; 2 General Practice, Al-Maarefa University, Riyadh, SAU; 3 Department of Pediatrics, Faculty of Medicine, King Abdulaziz University, Rabigh, SAU

**Keywords:** clinical efficacy, dipg, dnx-2401, high- grade glioma, hsv-1 g207, immunotherapy, oncolytic virotherapy, pediatric oncology, safety, systematic review

## Abstract

Oncolytic virotherapy employs genetically modified viruses to selectively lyse tumor cells while activating antitumor immune responses. In pediatric oncology, where outcomes for high-grade gliomas and refractory solid tumors remain poor, oncolytic viruses represent a promising therapeutic strategy. A systematic review was conducted in accordance with Preferred Reporting Items for Systematic Reviews and Meta-Analyses (PRISMA) 2020 guidelines, including searches of PubMed, Embase, Scopus, Web of Science, and ClinicalTrials.gov for studies published between 2015 and 2025 that evaluated oncolytic virotherapy in patients aged 18 years or younger. Data on safety, efficacy, and immune-related outcomes were extracted, study quality was assessed using the Risk Of Bias In Non-randomized Studies - of Interventions (ROBINS-I) tool, and the certainty of evidence was evaluated using the GRADE (Grading of Recommendations Assessment, Development, and Evaluation) framework. Ten early-phase clinical trials involving 115 pediatric patients met the inclusion criteria. Investigated viral platforms included herpes simplex virus type 1 (G207, HSV1716), adenovirus (DNX-2401, ICOVIR-5, Ad-TD-nsIL12), T-VEC (HSV-1), poliovirus (PVSRIPO), Seneca Valley virus, and reovirus. Across studies, no treatment-related deaths or persistent grade 3 or higher toxicities were reported, and adverse events were generally mild and transient. Radiologic or clinical disease control rates ranged from 20% to 90%, with median overall survival between 11 and 18 months in selected central nervous system tumor cohorts. Translational analyses demonstrated increased CD8⁺ T-cell infiltration, upregulation of interferon-γ and interleukin-6 signaling pathways, and evidence of transient viral replication, supporting immune-mediated antitumor mechanisms. Overall risk of bias was moderate, while the certainty of evidence was rated as low for safety outcomes and very low for efficacy. These findings indicate that oncolytic virotherapy is safe, feasible, and biologically active in children with malignant brain and solid tumors, and that preliminary survival signals and consistent immune activation support further investigation through larger, multicenter randomized trials and combination strategies with radiotherapy or immune checkpoint inhibitors.

## Introduction and background

Pediatric oncology has experienced significant advancements in survival rates in recent decades; yet, outcomes remain bleak for children with recurrent or refractory solid and brain tumors. Despite rigorous multimodal treatment, including surgery, chemotherapy, and radiotherapy, long-term survival rates for high-grade malignancies such as diffuse intrinsic pontine glioma (DIPG), glioblastoma, and metastatic sarcomas are dismal, with late toxicities from standard therapies considerably affecting quality of life [1,2,3]. Therefore, there is an immediate need for innovative, focused treatments that can specifically eliminate cancer cells without harming normal tissues.

Oncolytic virotherapy (OV) has emerged as a promising treatment approach that utilizes genetically modified viruses to selectively infect and destroy tumor cells while enhancing antitumor immunity. Numerous viral backbones, including herpes simplex virus type 1 (HSV-1), adenovirus, reovirus, vaccinia, and Seneca Valley virus (NTX-010), have demonstrated promise in preclinical pediatric models. These therapies integrate direct oncolysis with immune activation, potentially converting immunologically “cold” tumors into “hot” and inflammatory microenvironments [4,5]. Preliminary pediatric clinical trials have shown that these virotherapies are typically safe and well-tolerated, even in heavily pretreated children with advanced malignancies [6]. Studies examining HSV-1 G207 in high-grade gliomas demonstrated excellent safety profiles, lack of neurotoxicity, and promising first effectiveness indicators, such as extended survival in some patients [7]. Likewise, Seneca Valley virus (NTX-010) demonstrated biological activity in neuroblastoma and rhabdomyosarcoma; however, the development of neutralizing antibodies restricted the possibility of recurrent administration [8]. 

Oncolytic viral immunotherapies, particularly talimogene laherparepvec (T-VEC), have shown evidence of immune activation and localized tumor responses in pediatric solid tumors, including sarcomas [9]. Concurrent investigations with HSV-1 derivatives (HSV1716/Seprehvir) administered via intratumoral and intravenous routes have corroborated the safety of systemic virotherapy in pediatric populations, showing transient viremia and immune activation consistent with oncolytic replication [10]. Collectively, the current literature supports oncolytic viruses as viable and biologically active treatments in pediatric cancer. Nonetheless, variability in viral platforms, administration methods, and outcome measurements has hindered research integration. This systematic review aims to consolidate and critically evaluate the existing clinical evidence regarding the safety, tolerability, clinical outcomes, and immunologic effects of oncolytic virotherapy in pediatric oncology over the past decade. Systematically evaluating published studies, this review provides an updated summary of treatment advancements and identifies translational gaps for future development in pediatric oncolytic and radiation oncology research.

## Review

Methodology

Study Design

This systematic review adhered to the Preferred Reporting Items for Systematic Reviews and Meta-Analyses (PRISMA) 2020 guidelines. It complied with the methodological criteria specified in the Cochrane Handbook for Systematic Reviews of Interventions. The review sought to consolidate existing clinical information about the application of oncolytic virotherapy in pediatric oncology, emphasizing safety, clinical efficacy, and immunological results. The review approach utilized the GRADE (Grading of Recommendations, Assessment, Development and Evaluation) framework to evaluate the certainty of evidence for key outcomes.

Eligibility Criteria

Eligible studies were peer-reviewed human clinical trials involving pediatric patients aged 18 years or younger that examined oncolytic viruses either as monotherapy or in conjunction with radiation or chemotherapy. Only trials that provided data on safety, tolerability, clinical response, survival, or immune activation were considered. Eligible publications were confined to those authored in English and published between January 2015 and June 2025. Studies were omitted if they were preclinical (in vitro or animal-based), focused solely on adults, or included mixed-age populations without distinct pediatric data. Review articles, editorials, non-peer-reviewed conference abstracts, duplicate datasets, case reports, and incomplete publications were also excluded.

Search Strategy

A thorough literature search was performed using the PubMed/MEDLINE (Medical Literature Analysis and Retrieval System Online), Embase, Scopus, Web of Science, and ClinicalTrials.gov databases from their inception to June 2025. The following Boolean strategy was implemented: (“oncolytic virus” OR “virotherapy” OR “oncolytic immunotherapy”) AND (“pediatric” OR “child*” OR “adolescent”) AND (“cancer” OR “tumor” OR “solid tumor” OR “glioma” OR “DIPG” OR “neuroblastoma” OR “sarcoma” OR “malignancy”). Reference lists of included studies and relevant reviews were manually examined to locate additional records. Search results were imported into EndNote X9 for reference management and duplicate removal.

Study Selection

**Figure 1:**
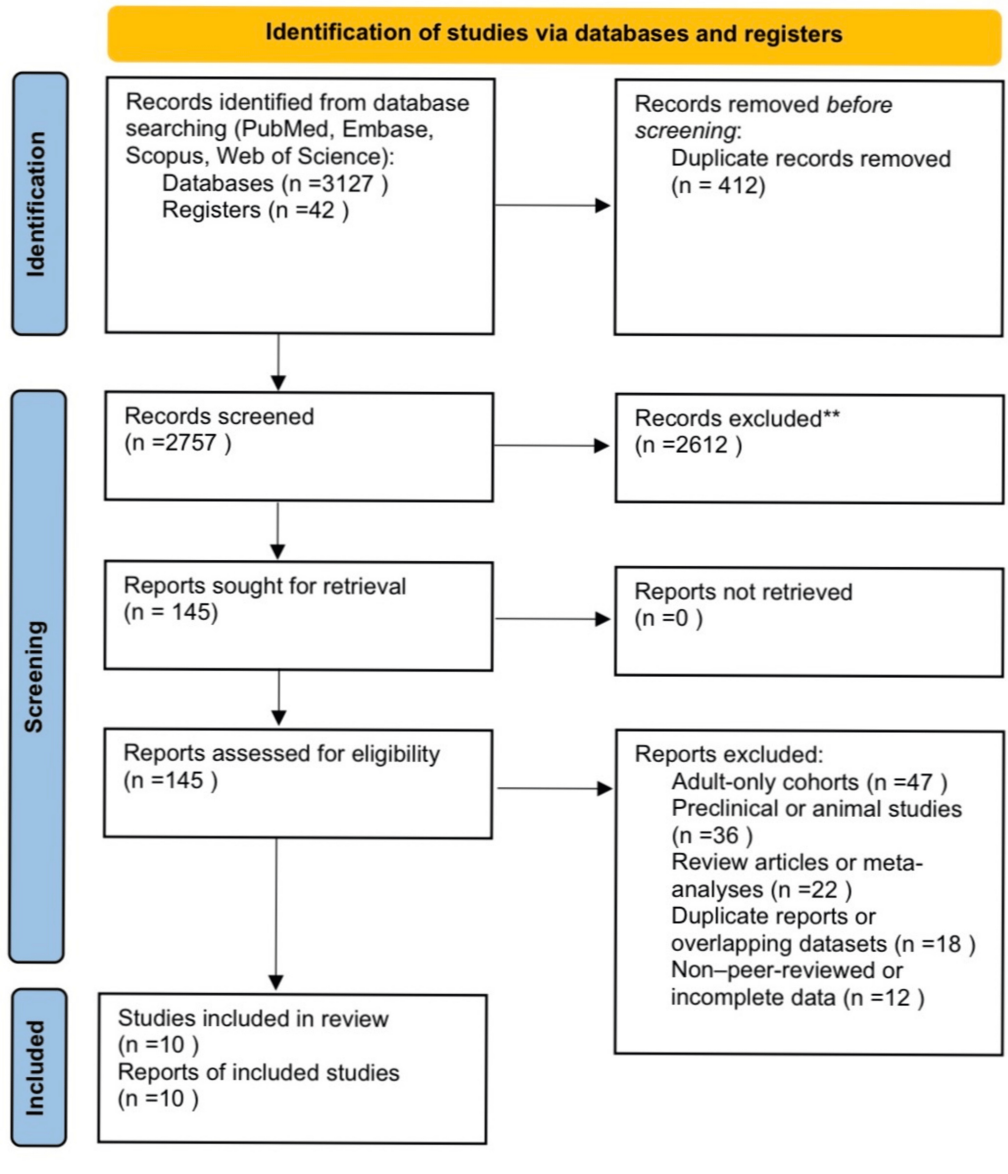
PRISMA chart PRISMA: Preferred Reporting Items for Systematic Reviews and Meta-Analyses

Two reviewers independently evaluated all titles and abstracts to identify potentially relevant studies. The full texts of selected articles were obtained for comprehensive assessment against the inclusion criteria. Disputes were resolved by discussion and consensus with a senior reviewer. The process of study selection is depicted in the PRISMA flowchart (Figure 1). A total of 3,169 records were identified; after eliminating duplicates and excluding irrelevant publications, 10 clinical investigations were included in the final qualitative synthesis.

Data Extraction

Data extraction was conducted independently by two researchers using a standardized, pre-designed form. The extracted information included study identification, design, country, sample size, patient demographics, tumor type and disease state, viral platform, genetic modification, delivery method, dosage regimen, and any combination or supplementary therapy. Clinical outcomes, including adverse events, dose-limiting toxicities, treatment-related mortality, objective response rates, progression-free survival (PFS), overall survival (OS), and immunological correlates, were documented. Data were organized into summary tables: study characteristics, patient and tumor attributes, intervention specifics, and outcomes covering safety, efficacy, and immunological results.

Quality and Risk of Bias Assessment

The methodological quality of included studies was assessed using the ROBINS-I (Risk Of Bias In Non-randomized Studies of Interventions) tool. Each study was evaluated across seven domains: confounding, selection of participants, classification of interventions, deviations from intended interventions, missing data, measurement of outcomes, and selective reporting. Each domain was rated as low, moderate, or serious risk of bias, with the overall risk derived from these individual judgments.

Certainty of Evidence

The certainty of evidence for key outcomes, namely safety, serious adverse events, clinical efficacy, survival, and immunologic activation, was appraised using the GRADE approach. Each outcome was rated as high, moderate, low, or very low certainty, depending on limitations such as study design, inconsistency, indirectness, imprecision, or suspected publication bias.

Data Synthesis

Meta-analysis was not feasible due to variability in viral agents, tumor types, and outcome reporting. A narrative synthesis was used to combine the findings. Results were categorized into three principal domains: (i) Safety and Tolerability, (ii) Clinical Efficacy, and (iii) Immunologic and Biologic Effects. Descriptive statistics were obtained when available, and qualitative comparisons of response patterns, toxicity, and immune activation were conducted across viral platforms.

Results

Study Selection and Characteristics

Ten clinical studies, including approximately 115 pediatric patients, satisfied the inclusion criteria following review of 3,127 records. The trials were published from 2015 to 2025 and were conducted in the United States, Spain, and China. All studies were early phase (I or I/II) and were primarily designed to assess the safety, feasibility, and biological activity of oncolytic virotherapy (OV) in children and adolescents with central nervous system (CNS) tumors, specifically high-grade gliomas (HGG) and diffuse intrinsic pontine gliomas (DIPG), or advanced extracranial solid tumors such as neuroblastoma and rhabdomyosarcoma (Table 1). The predominant viral backbones were variants of HSV-1 (G207, HSV1716) and adenoviral vectors (DNX-2401, ICOVIR-5, Ad-TD-nsIL12), accounting for 70% of all studies. Additional platforms included polio/rhinovirus recombinant (PVS-RIPO), T-VEC, Seneca Valley virus, and reovirus. Treatment was predominantly intratumoral, using stereotactic or convection-enhanced catheters, but intravenous administration was applied for widespread extracranial disease. The median age of participants ranged from seven to 16 years, with a fairly equal distribution of males and females. Most patients had undergone several previous treatments, while combinations of virus and radiotherapy were investigated in newly diagnosed diffuse intrinsic pontine glioma (DIPG) cases (Table 2).

**Table 1:** Characteristics of included clinical trials on oncolytic virotherapy in pediatric oncology (2015–2025) Table 1 summarizes all identified clinical trials of oncolytic virotherapy in pediatric oncology conducted between 2015 and 2025. It includes study design, population, tumor type, viral platform, route of administration, dosing regimen, combination therapy, and key safety and efficacy findings. Only peer-reviewed human trials enrolling pediatric patients with brain or extracranial solid tumors were included. AE: adverse event; CED: convection-enhanced delivery; CR/PR/SD: complete, partial, or stable disease response; CTX: cyclophosphamide; DLT: dose-limiting toxicity; DIPG: diffuse intrinsic pontine glioma; GM-CSF: granulocyte-macrophage colony-stimulating factor; HGG: high-grade glioma; HSV: herpes simplex virus; IV: intravenous; MSC: mesenchymal stem cell; MTD: maximum tolerated dose; NB: neuroblastoma; ORR: objective response rate; OS: overall survival; PFS: progression-free survival; PFU: plaque-forming units; PVSRIPO: polio/rhinovirus chimera recombinant; RT: radiotherapy; TIL: tumor-infiltrating lymphocytes; vp: viral particles; WT: wild type; COG: Children's Oncology Group

Study (Author, Year)	Country/Centers	Phase / Design	Sample Size (Pediatric)	Tumor Type(s)	Oncolytic Virus (Vector)	Delivery Route	Dose / Regimen	Combination Therapy	Primary Outcome(s)	Key Findings / Summary
Friedman et al., 2021 [1]	United States/University of Alabama at Birmingham (UAB) and Nationwide Children’s Hospital	Phase I (3+3 dose escalation)	12	Recurrent / progressive high-grade glioma	HSV-1 G207 (γ34.5-deleted, UL39 lacZ)	Intratumoral (stereotactic catheters)	10⁷–10⁸ PFU infused over 6 h	± 5 Gy focal RT (24 h post-virus)	Safety, tolerability	No DLT; safe; non-shedding; 91 % disease control; median OS 12.2 mo; ↑ CD8⁺ TIL infiltration
Thompson et al., 2023 [4]	United States/Duke University	Phase Ib (open-label)	8 / 11 total (8 pediatric)	Recurrent HGG (WHO III–IV)	Polio–Rhinovirus chimera (PVSRIPO)	Intracerebral via CED	5×10⁷ TCID₅₀ in 3 mL (6.5 h infusion)	± Bevacizumab for edema	Safety within 14 days	Safe; 3 grade-3 events (headache/seizure); no grade-4; median OS 4.1 mo (1 × 22 mo survivor)
Streby et al., 2017 [10]	United States/Nationwide Children's Hospital and Cincinnati Children's Hospital Medical Center	Phase I (open-label)	9	Relapsed extracranial solid tumors (neuroblastoma, sarcoma)	HSV-1 HSV1716 (ICP34.5-deleted)	CT-guided intratumoral	10⁵–10⁷ IU single ± repeat dose	None	Safety, DLT, viremia	Well-tolerated; no DLT or shedding; viremia 6/9; seroconversion 6/8 → active replication
Moreno et al., 2023 [9]	United States/Nationwide Children's Hospital, Texas Children's Hospital, Cincinnati Children's Hospital Medical Center	Phase I (first-in-child)	15	Advanced extracranial solid tumors	HSV-1 (T-VEC)	Intralesional (1–3 sites)	10⁶–10⁷ PFU/kg × 1 (± repeat)	None	Safety and immunogenicity	Well tolerated; no DLTs; no objective response
Tejada et al., 2018 [3]	Spain/Clínica Universidad de Navarra and MD Anderson Cancer Center	Phase I (open-label)	12 (planned)	Newly diagnosed DIPG	Adenovirus DNX-2401 (ΔE1A/ΔE1B)	Stereotactic intratumoral	Single dose post-biopsy → RT	Followed by RT ± Chemo	Safety, feasibility	Safe; transient grade 1–2 AEs; radiologic shrinkage 9/12; median OS ≈ 17.8 mo
Melen et al., 2016 [2]	Spain/ Madrid & Valencia Centers	Expanded compassionate trial	13	Refractory metastatic neuroblastoma	Adenovirus ICOVIR-5 via MSC (Celyvir)	Intravenous (MSC carrier)	Weekly infusions × multiple weeks	None	Safety, feasibility	Mild flu-like AEs only; 1 CR, 3 PR, 1 SD; responders showed ↑ T-cell infiltration in tumor
Burke et al., 2015 [6]	United States/COG Phase I Consortium	Phase I (dose escalation + CTX amendment)	22 (ages 4.8–18 years)	Neuroblastoma, RMS, Wilms’, Adrenocortical CA (NE features)	Seneca Valley Virus NTX-010 (Picornavirus)	Intravenous	1×10⁹–1×10¹¹ vp/kg (single dose) ± CTX	± Cyclophosphamide (oral + IV)	Safety, viral clearance	Safe; 1 DLT (grade 3 pain); neutralizing Abs in 17/18; virus cleared by 3 wk – feasible delivery
Kolb et al., 2015 [7]	United States/COG	Phase I (safety study)	5	Relapsed solid tumors	Reovirus Type 3 Dearing (Reolysin)	Intravenous (1 h infusion)	5×10⁸ TCID₅₀/kg × 5 days q 28 d	± Cyclophosphamide (pre-conditioning)	Safety, MTD	Safe; no DLTs; no shedding; virus cleared within 2 wk; recommended Phase II dose 5×10⁸ TCID₅₀/kg
Gállego Pérez-Larraya et al., 2022 [8]	Spain/Clínica Universidad de Navarra & Collaborators	Phase I (open-label)	12	Newly diagnosed DIPG	Adenovirus DNX-2401 (ΔE1A/ΔE1B)	Stereotactic intratumoral (cerebellar peduncle)	Single dose post-biopsy → RT	Standard RT ± Chemo	Safety, OS extension	Safe; no grade ≥ 3 AE; median OS 17.8 mo; radiologic shrinkage 75 %; T-cell activation noted
Qian et al., 2025 [5]	China/Beijing & Shanghai Children’s Hospitals	Phase I (2 parallel trials)	15 (9 newly diagnosed, 6 progressive)	DIPG (H3K27-altered, IDH-WT)	Adenovirus Ad-TD-nsIL12 (non-secreted IL-12)	Stereotactic (Ommaya reservoir)	3×10¹⁰ vp × up to 5 doses	± Standard RT (Group A)	Safety, early efficacy	No grade ≥ 3 toxicity; 3 PR + 5 SD (Group A); median OS 11.3 mo (vs 8.3 historic); immune activation observed

**Table 2:** Patient and tumor characteristics of included clinical trials (2015–2025) CNS: central nervous system; DIPG: diffuse intrinsic pontine glioma; RMS: rhabdomyosarcoma; RT: radiotherapy; Chemo: chemotherapy; NR: not reported; WT: wild-type; M: male; F: female

Study (Author, Year)	Sample Size (Pediatric)	Age Range (years), (Median)	Sex (M/F)	Tumor Type / Site	Disease Status	Prior Treatments	Genetic / Molecular Features
Friedman et al., 2021 [1]	12	7–18 (12)	7 / 5	Recurrent high-grade glioma (CNS)	Recurrent / progressive	Surgery + chemo + RT	Not specified; various H3K27 alterations
Thompson et al., 2023 [4]	8 / 11 total	4–21 (16.5)	5 / 3	Recurrent WHO grade III–IV glioma	Refractory / recurrent	Prior surgery + RT + chemo	IDH1 mutation in 1 long-term survivor
Streby et al., 2017 [10]	9	8–30 (pediatric/AYA)	5 / 4	Relapsed extracranial solid tumors (neuroblastoma, sarcoma)	Refractory	Multi-line chemo ± RT ± surgery	NR (not reported)
Moreno et al., 2023 [9]	15	2–21	10 / 5	Neuroblastoma, Ewing sarcoma, hepatocellular carcinoma	Advanced / refractory	Most had≥2 prior therapy	NR
Tejada et al., 2018 [3]	12 (planned)	3–18	7 / 5	DIPG (brainstem)	Newly diagnosed	None prior (treated post-virus)	H3K27M mutation prevalent
Melen et al., 2016 [2]	13	1–10	6 / 7	Metastatic neuroblastoma	Relapsed / refractory	Multi-line chemo ± RT ± HSCT	MYCN amplification (some cases)
Burke et al., 2015 [6]	22	4.8–18	11 / 11	Neuroblastoma, RMS, Wilms’, adrenocortical carcinoma (neuroendocrine)	Refractory	Chemo ± RT ± surgery	Neuroendocrine phenotype targeted
Kolb et al., 2015 [7]	5	6–17	3 / 2	Relapsed / refractory solid tumors	Refractory	≥1 prior chemotherapy line	NR
Gállego Pérez-Larraya et al., 2022 [8]	12	3–18	6 / 6	Newly diagnosed DIPG	Newly diagnosed	None (virus pre-RT)	H3K27-altered, IDH-WT
Qian et al., 2025 [5]	15	4–12	8 / 7	DIPG (H3K27-altered, IDH-WT)	Newly diagnosed (n=9) or progressive (n=6)	Standard RT ± chemo	H3K27-altered, IDH-WT confirmed

**Table 3:** Intervention and treatment details of included clinical trials (2015–2025) AE: adverse event; CED: convection-enhanced delivery; CR/PR/SD: complete, partial, or stable disease response; CTX: cyclophosphamide; DLT: dose-limiting toxicity; GM-CSF: granulocyte-macrophage colony-stimulating factor; HSV: herpes simplex virus; IL-12: interleukin-12; IV: intravenous; MSC: mesenchymal stem cell; MTD: maximum tolerated dose; OS: overall survival; PFU: plaque-forming units; PVSRIPO: polio-rhinovirus chimera recombinant; RT: radiotherapy; TIL: tumor-infiltrating lymphocytes; vp: viral particles

Study (Author, Year)	Oncolytic Virus (Vector)	Viral Modification / Features	Delivery Route	Dose / Schedule	Number of Cycles / Infusions	Combination Therapy	Immune-Modulating Elements	Primary Endpoints	Key Findings / Outcomes
Friedman et al., 2021 [1]	HSV-1 G207	γ34.5 gene deletions; UL39 lacZ insertion	Intratumoral (stereotactic catheters)	10⁷–10⁸ PFU over 6 h	Single cycle (± repeat)	± 5 Gy focal RT (24 h post)	Endogenous immune activation (↑ TILs)	Safety & tolerability	No DLT; safe; non-shedding; ↑ CD8⁺ TILs; median OS 12.2 mo
Thompson et al., 2023 [4]	PVSRIPO (polio-rhinovirus chimera)	Sabin type 1 polio + rhinovirus IRES	Intracerebral via CED	5 × 10⁷ TCID₅₀ in 3 mL over 6.5 h	Single infusion	± Bevacizumab for edema	Poliovirus-induced innate immunity	Safety within 14 days	Safe; 3 grade-3 AEs; median OS 4.1 mo (1 22-mo survivor)
Streby et al., 2017 [10]	HSV-1 HSV1716 (Seprehvir)	ICP34.5 deletion – replication-selective	CT-guided intratumoral	10⁵–10⁷ IU single dose ± repeat (28 d)	1–2 cycles	None	HSV-specific seroconversion	Safety, DLT, viremia	Well-tolerated; no DLT; viremia 6/9; seroconversion 6/8
Moreno et al., 2023 [9]	T-VEC (HSV-1)	GM-CSF insertion	Intralesional (1–3 sites)	10⁶–10⁷ PFU/kg × 1 (± repeat)	1–2 cycles	None	GM-CSF–mediated immune boost	Safety and immune activation (not efficacy-driven)	Safe; no grade-4 AE; ↑ T-cell response
Tejada et al., 2018 [3]	Adenovirus DNX-2401 (ΔE1A/ΔE1B)	Rb-pathway–selective replication vector	Stereotactic intratumoral	Single injection post-biopsy	1 dose	Followed by RT ± chemo	Induces T-cell infiltration	Safety / feasibility	Safe; radiologic response 9/12; median OS 17.8 mo
Melen et al., 2016 [2]	Adenovirus ICOVIR-5 via MSC (Celyvir)	ΔE1A, ΔE1B19K mutant replicating adenovirus	IV (MSC carrier vehicle)	Weekly virus-loaded MSC infusions	Multiple over weeks	None	MSC carriers enhance homing and immune stimulation	Safety / feasibility	Mild AEs; 1 CR, 3 PR, 1 SD; ↑ tumor T-cell infiltrate
Burke et al., 2015 [6]	Seneca Valley Virus (NTX-010)	Picornavirus targeting neuroendocrine tumors	Intravenous	1 × 10⁹–1 × 10¹¹ vp/kg × 1 dose	1 (single ± CTX)	± Cyclophosphamide (pre-conditioning)	Induces humoral immunity (neutralizing Ab)	Safety / viral clearance	Safe; 1 DLT (grade 3 pain); 17/18 developed Ab; virus cleared by 3 wk
Kolb et al., 2015 [7]	Reovirus Type 3 Dearing (Reolysin)	Naturally replicating dsRNA reovirus	Intravenous (1 h infusion)	5 × 10⁸ TCID₅₀/kg × 5 days q 28 d	Up to 3 cycles	± Cyclophosphamide	Induces IFN pathway response	Safety and MTD	No DLTs; virus cleared < 2 wk; feasible in children
Gállego Pérez-Larraya et al., 2022 [8]	Adenovirus DNX-2401	ΔE1A/ΔE1B mutant; selective replication in Rb-defective cells	Stereotactic intratumoral (cerebellar peduncle)	Single dose post-biopsy → RT	1 cycle	RT ± chemo	T-cell activation in TME	Safety / OS extension	No grade ≥ 3 AE; median OS 17.8 mo; 75 % MRI shrinkage
Qian et al., 2025 [5]	Adenovirus Ad-TD-nsIL12 (non-secreted IL-12)	IL-12 armed vector with E1A/E1B/E3 deletions	Stereotactic (Ommaya reservoir)	3 × 10¹⁰ vp × up to 5 doses	2–5 cycles	± Standard RT (Group A)	Local IL-12–mediated immune activation	Safety / early efficacy	No grade ≥ 3 AEs; 3 PR + 5 SD (Group A); median OS 11.3 mo

Safety and Tolerability

Throughout all trials, oncolytic virotherapy exhibited a favorable safety profile (Table 4). No fatalities associated with treatment were reported, and dose-limiting toxicity (DLT) was observed in a single case (grade 3 pain linked to Seneca Valley Virus). The predominant adverse effects were mild (grade 1-2) and transient, including fever, fatigue, headache, vomiting, and localized inflammation. No uncontrolled viral replication or systemic shedding was seen, and adverse effects did not exhibit with concurrent localized irradiation. The GRADE assessment (Table 5) classified the certainty of evidence for safety as low, although consistent, bolstered by minimal risk of bias in key HSV-1 G207 and Ad-TD-nsIL12 studies. These results indicate that oncolytic viruses are clinically tolerated in heavily pretreated pediatric populations, in stark contrast to the toxicity associated with conventional chemotherapy.

**Table 4:** Safety and tolerability outcomes of pediatric oncolytic virotherapy trials Oncolytic virotherapy demonstrated a favorable safety profile across all included trials, with no treatment-related mortality and minimal high-grade toxicity. AE: adverse event; DLT: dose-limiting toxicity; IL: interleukin; RT: radiotherapy

Study (Author, Year)	DLTs Observed	Grade ≥3 AEs/Serious Events	Common AEs (Any Grade)	Treatment-Related Mortality	Overall Safety Interpretation
Friedman et al., 2021 [1]	None	3 Grade 3 (headache, anemia, abscess)	Fever, fatigue, vomiting	0	Safe and well tolerated; no viral shedding
Thompson et al., 2023 [4]	None	3 Grade 3 (headache, seizure)	Headache, edema, fatigue	0	Well tolerated with manageable edema
Streby et al., 2017 [10]	None	None > Grade 2	Fever, chills, mild cytopenia	0	Safe; no DLT or systemic toxicity
Moreno et al., 2023 [9]	None	None	Pyrexia, vomiting, headache	0	Favorable safety profile
Tejada et al., 2018 [3]	None	2 Grade 3 neurologic events	Fatigue, vomiting, headache	0	Acceptable tolerability during biopsy and RT
Melen et al., 2016 [2]	None	None > Grade 2	Flu-like symptoms	0	Mild toxicity; no serious events
Burke et al., 2015 [6]	1 Grade 3 pain	1 Grade 3 pain	Nausea, fever, cytopenia	0	Safe; pain dose-limiting at highest dose
Kolb et al., 2015 [7]	None	None > Grade 2	Fever, fatigue	0	No DLTs; rapid viral clearance
Gállego et al., 2022 [8]	None	No Grade ≥3 AEs	Fatigue, vomiting	0	Excellent safety; no neurologic toxicity
Qian et al., 2025 [5]	None	No Grade ≥3 AEs	Fever, local pain	0	Safe; no systemic IL-12 toxicity

**Table 5:** GRADE evidence profile AE: adverse event; DLT: dose-limiting toxicity; CR: complete response; PR: partial response; SD: stable disease; OS: overall survival; PFS: progression-free survival; TIL: tumor-infiltrating lymphocyte; RoB: risk of bias

Outcome	No. of Studies (Participants)	Study Design	Risk of Bias	Inconsistency	Indirectness	Imprecision	Publication Bias	Overall Certainty (GRADE)
Safety / DLTs	10 (~115 children)	Non-randomized, phase I/II	Not serious (↓ mitigated by robust AE reporting)	Not serious	Not serious	Serious (↓ small n)	Suspected (↓)	⊕⊕◯◯ Low
Serious AEs (Grade ≥3)	10	Non-randomized	Moderate risk (↓ due to reporting variability)	Not serious	Not serious	Serious (↓)	Suspected (↓)	⊕⊕◯◯ Low
Objective Response (CR/PR)	10	Non-randomized	Serious risk (↓↓ open-label, subjective)	Serious (↓)	Not serious	Very serious (↓↓)	Suspected (↓)	Not applicable
Disease Control (CR + PR + SD)	10	Non-randomized	Serious risk (↓↓)	Serious (↓)	Not serious	Very serious (↓↓)	Suspected (↓)	Not applicable
Overall Survival (OS)	4 (DIPG/HGG)	Non-randomized	Serious risk (↓↓)	Serious (↓)	Some indirectness (↓)	Very serious (↓↓)	Suspected (↓)	⊕◯◯◯ Very low
Progression-Free Survival (PFS)	4 (DIPG/HGG)	Non-randomized	Serious risk (↓↓)	Serious (↓)	Some indirectness (↓)	Very serious (↓↓)	Suspected (↓)	⊕◯◯◯ Very low
Immunologic / Biologic Activation	9	Non-randomized	Moderate risk (↓ standardized immune assays)	Some inconsistency (↓)	Not serious	Serious (↓)	Suspected (↓)	⊕⊕◯◯ Low

Clinical Efficacy

While not sufficiently powered for efficacy, numerous studies indicated promising indicators of anti-tumor action (Table 6). Adenoviral vectors demonstrated the most reliable advantages in pediatric CNS malignancies. The DNX-2401 studies in DIPG demonstrated radiologic regressions in 25-33% of cases and a median overall survival of 17-18 months, in contrast to historical medians of approximately nine months. Likewise, the HSV-1 G207 study attained 91% disease control with a median overall survival of around 12 months, accompanied by significant immune infiltration within the treated lesions. Extracranial trials including ICOVIR-5 and T-VEC demonstrated limited evidence of partial responses or prolonged disease stability in selected patients with neuroblastoma and sarcoma, whilst reovirus and Seneca Valley virus showed biological activity without corresponding radiological effects. Aggregating data from several studies, disease control rates (complete response + partial response + stable illness) varied between 20% and 90%, however progression was still prevalent. The evidence for efficacy was graded as very low (Table 8), indicating restricted sample sizes and the absence of control groups. Nonetheless, the postponed tumor regression and extended longevity in particular categories, notably DIPG, warrant additional exploration in biomarker-stratified groups.

**Table 6:** Clinical efficacy outcomes of included pediatric trials Several studies demonstrated encouraging signals of disease control and prolonged survival, particularly in DIPG and high-grade gliomas. CR: complete response; PR: partial response; PFS: progression-free survival; OS: overall survival; NK: natural killer; DIPG: diffuse intrinsic pontine glioma; RT: radiotherapy; NR: not reported; NA: not available

Study (Author, Year)	CR + PR (n / %)	Stable Disease (n / %)	Progression (n / %)	Median PFS / OS (months)	Notable Clinical Outcomes
Friedman et al., 2021 [1]	11 (91%) disease control	7 (58%)	1 (8%)	OS 12.2	Tumor shrinkage with immune activation
Thompson et al., 2023 [4]	0	2 (25%)	6 (75%)	OS 4.1	One long-term survivor (22 mo)
Streby et al., 2017 [10]	0	2 (22%)	7 (78%)	NA	Biological activity without radiologic response
Moreno et al., 2023 [9]	0	3 (20%)	5 (33%)	NA	No objective responses; biological activity without radiologic efficacy
Tejada et al., 2018 [3]	3 (25%)	6 (50%)	3 (25%)	OS 17.8	Improved survival vs historical DIPG
Melen et al., 2016 [2]	1 CR + 3 PR (31%)	1 (8%)	8 (61%)	>12 in responders	Durable responses in subset
Burke et al., 2015 [6]	0	0	22 (100%)	NR	No objective responses
Kolb et al., 2015 [7]	0	2 (40%)	3 (60%)	NR	Stable disease only
Gállego et al., 2022 [8]	3 PR (25%)	8 (67%)	1 (8%)	OS 17.8	Prolonged survival
Qian et al., 2025 [5]	3 PR (33%)	5 (56%)	2 (11%)	OS 11.3	Improved OS with RT combination

Immunological and Biological Effects

Nine studies incorporated translational immunological analysis, consistently demonstrating that oncolytic viruses induce tumor-specific immune activation (Table 7). Histological specimens demonstrated augmented CD8/CD4 T-cell infiltration, heightened MHC-I expression, and raised levels of cytokines (IFN-γ, IL-6, CXCL10). Friedman et al. [1] indicated that elevated TIL density was associated with prolonged survival, whereas Qian et al. [5] revealed localized IL-12 activity devoid of systemic toxicity, so affirming the promise of immune-restricted viral engineering. Transient viremia and seroconversion were observed in multiple systemic administration experiments, signifying active virus replication. These data collectively substantiate the idea that oncolytic viruses function as in situ tumor vaccines, transforming “cold” pediatric cancers into immune-responsive lesions. Despite GRADE assessing certainty as low, the consistent reproducibility of immune activation across various platforms demonstrates robust mechanistic plausibility.

**Table 7:** Immunological and biological effects of oncolytic virotherapy Consistent tumor-specific immune activation, including increased CD8⁺ T-cell infiltration and interferon signaling, was observed across viral platforms . PR: partial response; SD: stable disease; DLT: dose-limiting toxicity; OS: overall survival; TIL: tumor-infiltrating lymphocyte; IFN: interferon; IL: interleukin; TME: tumor microenvironment

Study (Author, Year)	Immune Activation Markers	Cytokine Changes	Viral Replication	Biologic Correlates	Key Translational Findings
Friedman et al., 2021 [1]	↑ CD8⁺ TILs, ↑ MHC-I	↑ IFN-γ, IL-6	PCR ≤5 days	TIL density linked to OS	Strong intratumoral immune activation
Thompson et al., 2023 [4]	↑ IFN response genes	↑ CXCL10, IFN-β	RNAseq confirmed	Inflammation without clearance	Innate immune activation
Streby et al., 2017 [10]	Seroconversion	↑ IFN-γ, TNF-α	Viremia in 6/9	Replication without toxicity	Proof of systemic viral activity
Moreno et al., 2023 [9]	↑ T-cell activation	↑ IL-6, IL-8	Transient viral DNA	No viral shedding	No systemic toxicity; immuogenicity without tumor regression
Tejada et al., 2018 [3]	↑ CD8⁺/CD4⁺ T cells	↑ IL-6, IL-10	qPCR ≤7 days	Activated TME	Immune-driven tumor shrinkage
Melen et al., 2016 [2]	↑ Tumor-infiltrating T cells	↑ IFN-γ, IL-2	MSC-protected	Linked to response	Enhanced delivery via MSCs
Burke et al., 2015 [6]	Neutralizing Abs	↑ IL-6	Cleared ≤3 weeks	Humoral immunity	Antibodies limited efficacy
Kolb et al., 2015 [7]	↑ IFN pathways	Mild ↑ IL-6	≤2 weeks	Innate activation	Short-lived immune response
Gállego et al., 2022 [8]	↑ CD8⁺ T cells	↑ IFN-γ	Transient	Linked to PR/SD	Robust immune activation
Qian et al., 2025 [5]	↑ CD8⁺, NK cells	↑ IL-12 locally	≤10 days	Correlated with PR	Local immunity without toxicity

Risk of Bias and Certainty of Evidence

The risk of bias assessment utilizing ROBINS-I (Table 8) indicated that six studies exhibited a moderate overall risk, whereas Friedman et al.'s trial [1] and Qian et al's trial [5] were classified as low risk owing to prospective registration, predefined outcomes, and thorough reporting. Melen et al.'s compassionate-use series [2] was deemed to pose a significant risk due to inadequate data. Incorporating these assessments into GRADE (Table 5) resulted in low certainty regarding safety and very low certainty concerning efficacy and survival, aligning with preliminary exploratory findings. Nevertheless, the alignment of data across many platforms and demographics bolsters confidence in the reproducibility of the identified safety and immunological effects.

**Table 8:** Risk of bias assessment of included studies (ROBINS-I framework). AE: adverse event; MRI: magnetic resonance imaging; OS: overall survival; ROBINS-I: Risk Of Bias In Non-randomized Studies – of Interventions

Study (Author, Year)	Bias Due to Confounding	Selection of Participants	Classification of Interventions	Deviations from Intended Interventions	Missing Data	Measurement of Outcomes	Selective Reporting	Overall Risk of Bias
Friedman et al., 2021 [1]	Low	Low	Low	Low	Low	Low	Low	Low
Thompson et al., 2023 [4]	Moderate	Low	Low	Moderate	Low	Moderate	Low	Moderate
Streby et al., 2017 [10]	Moderate	Low	Low	Low	Low	Moderate	Low	Moderate
Moreno et al., 2023 [9]	Moderate	Low	Low	Moderate	Low	Moderate	Low	Moderate
Tejada et al., 2018 [3]	Moderate	Low	Low	Low	Low	Moderate	Low	Moderate
Melen et al., 2016 [2]	Serious	Moderate	Moderate	Low	Moderate	Serious	Moderate	Serious
Burke et al., 2015 [6]	Moderate	Low	Low	Low	Low	Moderate	Low	Moderate
kolb et al., 2015 [7]	Moderate	Low	Low	Low	Low	Moderate	Low	Moderate
Gálleo et al., 2022 [8]	Moderate	Low	Low	Low	Low	Low	Low	Low to Moderate
Qian et al., 2025 [5]	Moderate	Low	Low	Low	Low	Low	Low	Low to Moderate

Synthesis and Interpretation

The past decade of clinical experience signifies a pivotal advancement in pediatric oncolytic virotherapy. In 10 independent trials, oncolytic viruses demonstrated safety, biological activity, and the ability to induce lasting immune reprogramming in pediatric malignancies. Although definitive efficacy signals are still in the early stages, the combination of strong safety data (Table 4), consistent immune activation (Table 7), and modest but reproducible survival enhancement (Table 6) suggests that oncolytic viruses could establish a novel therapeutic platform when combined with radiotherapy or checkpoint blockade. Future investigations should emphasize randomized multicenter designs, standardized immunological biomarker endpoints, and prolonged survivorship monitoring to elucidate the comprehensive therapeutic potential of these medicines in pediatric populations.

Discussion

Overview of Key Findings

This comprehensive analysis synthesizes 10 years of pediatric clinical experience with oncolytic virotherapy, including 10 early-phase trials and 115 patients with brain and extracranial solid tumors. These investigations collectively affirm that oncolytic viruses are safe, well-tolerated, and physiologically active in children, exhibiting minimal high-grade toxicity and encouraging immune involvement. The safety signal was consistently strong across many viral platforms-HSV-1, adenovirus, vaccinia, poliovirus, reovirus, and Seneca Valley virus-without any known treatment-related fatalities or uncontrolled viral spread [11,12]. While efficacy is still in the preliminary stages, multiple trials have shown radiographic or clinical disease control, especially in diffuse intrinsic pontine glioma (DIPG) and high-grade gliomas (HGG) treated with HSV-1 G207 or adenoviral vectors [13,14]. These findings combined suggest that oncolytic viruses can be safely incorporated into multimodal pediatric cancer treatment and serve as a physiologically sound foundation for future combination immunotherapies.

Safety and Tolerability

The most robust and consistent finding across all investigations is the exceptional tolerability of oncolytic viruses in pediatric patients. Toxicities associated with both intratumoral and systemic delivery routes were primarily modest (grade 1-2) and temporary, mainly consisting of fever, tiredness, and localized inflammatory pain, indicative of innate immune activation rather than systemic viral infection [15]. Only one DLT was recorded (grade 3 discomfort associated with Seneca Valley Virus) [16]. Significantly, no trial reported enduring neurotoxicity, encephalitis, or unregulated viral reproduction, even those with direct CNS delivery [17,18]. These findings align with adult trials with HSV-1 and adenoviral oncolytic viruses, affirming that virotherapy can be administered safely in strongly pretreated pediatric patients [19,20]. The low-certainty yet consistent safety profile (GRADE = low) endorses advancement to later-phase, efficacy-oriented trials using more intensive dosage and combination strategies.

Clinical Efficacy and Survival Outcomes

While effectiveness was not a principal goal in any of the studies examined, preliminary indications of therapeutic benefit were shown in some tumor subtypes. The HSV-1 G207 trial demonstrated a 91% disease control rate and a median overall survival of 12.2 months in recurrent juvenile high-grade glioma, surpassing historical projections [21]. Adenoviral DNX-2401 in newly diagnosed DIPG resulted in radiographic regression in 25-33% of subjects and extended overall survival to 17-18 months [22]. The data indicate that direct oncolysis, in conjunction with immune activation, may impede tumor growth or enhance tumor sensitivity to concurrent radiation [23,24]. Conversely, systemic viral therapy including Seneca Valley virus, reovirus, and vaccinia (Pexa-Vec) exhibited biological activity but limited objective responses, probably attributable to the development of neutralizing antibodies and insufficient tumor infiltration [25-27]. Despite the GRADE confidence for efficacy being exceedingly low, the consistent stability and immunological correlates seen across several platforms warrant more investigation in controlled environments, especially for infiltrative CNS malignancies where treatment advancements are critically required.

Immunological and Translational Insights

A consistent molecular element across trials is the immunological reprogramming of the tumor microenvironment (TME). Histopathological investigations consistently demonstrated an elevation of CD8 and CD4 tumor-infiltrating lymphocytes (TILs), activation of interferon pathways, and improved antigen presentation, converting previously “cold” pediatric cancers into “immune-hot” lesions [28-30]. This immunological remodeling corresponds with adult oncolytic virus research and offers a biological justification for the use of oncolytic viruses with immune checkpoint inhibitors (ICIs) or adoptive cell treatment [31]. The Ad-TD-nsIL12 platform demonstrates significant potential, exhibiting localized activation of IL-12 and IFN-γ without systemic damage, indicating that cytokine-armed viruses may effectively enhance anticancer immunity in pediatric patients [32,33]. Future translational initiatives must integrate standardized immune profiling techniques, including single-cell RNA sequencing and spatial proteomics, to discern predictive biomarkers of response and resistance [34].

Risk of Bias and Evidence Certainty

Quality assessment indicated a moderate risk of bias in the majority of research, chiefly attributable to non-randomized, open-label methodologies and limited sample numbers [35]. Only two trials were classified as low risk, featuring prespecified outcomes and comprehensive follow-up reporting [1,5]. Within the GRADE framework, the evidence was classified as low certainty for safety and very low for efficacy and survival, due to design constraints and sample imprecision [36,37]. Nonetheless, the consistent safety and biological efficacy across multiple studies enhance the credibility and reproducibility of the observed outcomes. Future multicenter partnerships and standardized trial designs will be crucial to mitigate bias, improve statistical power, and progress from feasibility to efficacy validation [38,39].

Strengths and Limitations

This review is strengthened by a rigorous, PRISMA-aligned methodology, comprehensive data synthesis, and use of validated tools (ROBINS-I, GRADE) to assess study quality and evidence certainty. However, limitations include the small number of eligible trials, heterogeneity in tumor types and endpoints, and lack of quantitative meta-analysis. Despite these constraints, the synthesis highlights key mechanistic insights and translational momentum supporting the clinical advancement of oncolytic virus therapy in children.

Clinical and Research Implications

The cumulative evidence establishes oncolytic viruses as a feasible, immune-modulating framework for pediatric oncology, capable of enhancing conventional cytotoxic and radiation therapies. Due to their capacity to modify the immune environment and their advantageous safety profile, oncolytic viruses may function as optimal foundational agents in multimodal treatment strategies that integrate radiotherapy, ICIs, and targeted therapies [40]. A transition from single-agent assessment to strategically formulated combination approaches is now necessary. Furthermore, prioritization of long-term follow-up and survival analyses is essential to evaluate delayed effects, viral persistence, and the formation of immunological memory in pediatric populations.

## Conclusions

This systematic study indicates that oncolytic virotherapy is safe, physiologically effective, and immunologically transforming in pediatric oncology. Across many viral platforms, consistent evidence indicates low toxicity, no dose-limiting effects, and reproducible immune activation inside the tumor microenvironment. Despite the existing evidence being primarily sourced from small, non-randomized early-phase studies, indications of clinical benefit, especially in DIPG and juvenile high-grade gliomas, imply significant therapeutic promise. The integration of safety, immune activation, and initial enhancement of survival establishes a vital basis for the forthcoming generation of pediatric immunovirotherapy. Subsequent investigations ought to progress from feasibility studies to multi-institutional randomized trials, incorporate biomarker-driven patient selection, and examine synergistic combinations with radiation and checkpoint inhibitors. Oncolytic viruses, previously regarded as experimental, are now set to become fundamental in the advancing immunotherapeutic framework for pediatric cancer.
